# Combination of radiotherapy and vaccination overcomes checkpoint blockade resistance

**DOI:** 10.18632/oncotarget.9915

**Published:** 2016-06-07

**Authors:** Wenxin Zheng, Kinga B. Skowron, Jukes P. Namm, Byron Burnette, Christian Fernandez, Ainhoa Arina, Hua Liang, Michael T. Spiotto, Mitchell C. Posner, Yang-Xin Fu, Ralph R. Weichselbaum

**Affiliations:** ^1^ Department of Radiation and Cellular Oncology, University of Chicago, Chicago, IL, USA; ^2^ Department of Surgery, University of Chicago, Chicago, IL, USA; ^3^ The Ludwig Center for Metastasis Research, University of Chicago, Chicago, IL, USA; ^4^ Department of Surgery, Loma Linda University Health, Loma Linda, CA, USA; ^5^ Department of Pathology, UT Southwestern Medical Center, Dallas, TX, USA

**Keywords:** checkpoint blockade, T cell infiltration, radiation therapy, vaccination, tumor model

## Abstract

The majority of cancer patients respond poorly to either vaccine or checkpoint blockade, and even to the combination of both. They are often resistant to high doses of radiation therapy as well. We examined prognostic markers of immune cell infiltration in pancreatic cancer. Patients with low CD8^+^ T cell infiltration and high PD-L1 expression (CD8^+^ T^lo^PD-L1^hi^) experienced poor outcomes. We developed a mouse tumor fragment model with a trackable model antigen (SIYRYYGL or SIY) to mimic CD8^+^ T^lo^PD-L1^hi^ cancers. Tumors arising from fragments contained few T cells, even after vaccination. Fragment tumors responded poorly to PD-L1 blockade, SIY vaccination or radiation individually. By contrast, local ionizing radiation coupled with vaccination increased CD8^+^ T cell infiltration that was associated with upregulation of CXCL10 and CCL5 chemokines in the tumor, but demonstrated modest inhibition of tumor growth. The addition of an anti-PD-L1 antibody enhanced the effector function of tumor-infiltrating T cells, leading to significantly improved tumor regression and increased survival compared to vaccination and radiation. These results indicate that sequential combination of radiation, vaccination and checkpoint blockade converts non-T cell-inflamed cancers to T cell-inflamed cancers, and mediates regression of established pancreatic tumors with an initial CD8^+^ T^lo^PD-L1^hi^ phenotype. This study has opened a new strategy for shifting “cold” to hot tumors that will respond to immunotherapy.

## INTRODUCTION

Despite the emergence of immune checkpoint blockade as a promising new treatment for cancers, a majority of cancer patients, including pancreatic cancer patients, do not benefit from monotherapy with PD-L1 or CTLA-4 blockade [1, 2]. The lack of benefit from checkpoint inhibitors in pancreatic cancer patients parallels the lack of response to these agents in non-T cell-inflamed melanomas and other cancers bearing a phenotype of minimal T cell infiltration [3–5]. In addition to the lack of response to checkpoint inhibitors, non-T cell-inflamed melanomas are also characterized by the downregulation of chemokines and other immune markers [5]. Consequently, it remains unclear what additional modalities are required to facilitate responses to checkpoint inhibitors and other immunotherapies for these immunoresistant types of cancers.

Radiation therapy is widely used to treat cancer. The effects of radiation are proposed to be due mainly to its genotoxic effect via the induction of DNA damage and direct killing of tumor cells, but its role in the treatment of localized pancreatic cancer is currently debated. Although some data suggests that radiotherapy improves local control and increases resection rates in locally advanced tumors, other trials suggest that radiotherapy does not improve survival in pancreatic cancer [6–8]. In recent years, studies from our group and others have demonstrated that ionizing radiation (IR) functions as an immune modulator and regulates both the innate and adaptive immune systems [9–12]. IR regulates several processes of the immune response, including production of inflammatory cytokines/chemokines, antigen exposure and presentation, T cell priming, as well as regulation of immune-suppressive cells and factors [9, 10]. It was also proposed that IR induces immunological cell death, which may release tumor antigen and thus serve as an *in situ* vaccine to induce T cell priming [9, 10]. However, the significance of such *in situ* priming for tumor control remains to be further verified both in laboratory models and in clinical applications.

Here, we sought to identify immunological features in pancreatic cancers that predicted worse outcomes for patients and identified the combination of low CD8^+^ T cell infiltration and high PD-L1 expression (CD8^+^ T^lo^PD- L1^hi^) as an adverse prognostic feature. These non-T cell-inflamed (“cold”) tumors in our model respond poorly to immunotherapies involving antigen-specific vaccination or PD-L1 blockade. By contrast, IR coupled with vaccination induced a T cell-inflamed microenvironment that then overcame anti-PD-L1 resistance. Our results provide a step-by-step strategy to break tumor immune barriers in aggressive tumors by converting a non-T cell-inflamed phenotype to a T cell-inflamed phenotype that leads to tumor regression.

## RESULTS

### Low CD8^+^ T cell infiltration and high PD-L1 expression predicts worse survival in pancreatic cancer patients

**Figure 1:**
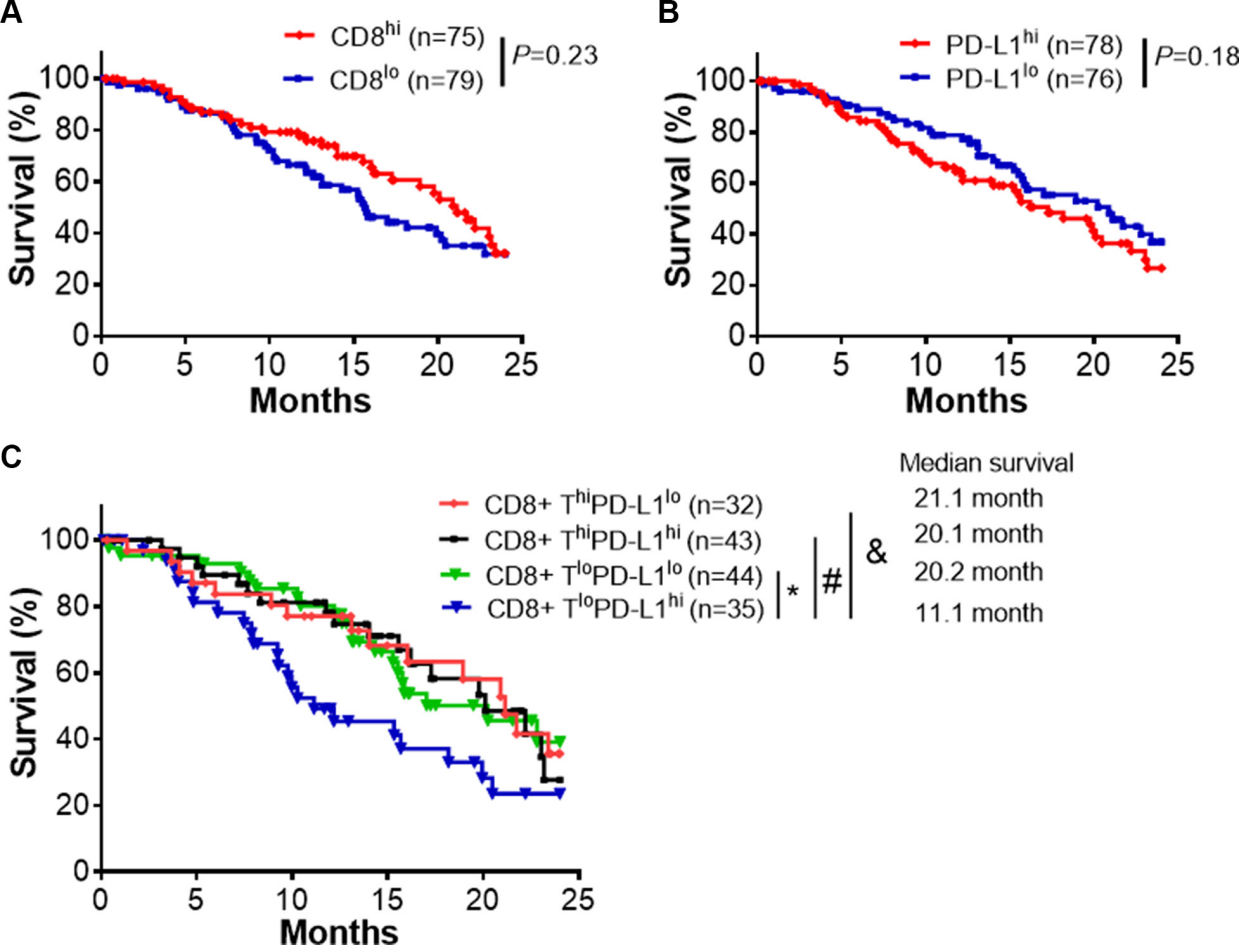
CD8^+^ T cell infiltrates and PD-L1 expression predict clinical outcomes (**A**) Survival analysis of pancreatic cancer patients (TCGA database) with high (CD8^+^ T^hi^) and low (CD8^+^ T^lo^) infiltration of CD8^+^ T cells. The patients were split into two groups by the median of CD8^+^ T percentage. (**B**) Survival analysis of the available pancreatic cancer patient cohort with high (PD-L1^hi^) and low (PD- L1^lo^) expression of PD-L1. (**C**) Survival analysis of pancreatic cancer patient cohorts with indicated level of CD8^+^ T infiltrates and PD-L1 expression. The high and low level of CD8^+^ T infiltrates or PD-L1 expression were defined by their comparison to the median of CD8^+^ T percentage and the median of overall PD-L1 expression. The percentage of CD8^+^ T cells were predicted by CIBERSORT using the gene expression data from TCGA database (Details in Materials and Methods). *P = 0.039, ^#^*P* = 0.064, & *P* = 0.066 (Mantel-Cox test).

We estimated CD8^+^ T cell infiltration using gene expression profiling in 183 pancreatic cancer specimens from The Cancer Genome Atlas (TCGA). To achieve this estimate, we used CIBERSORT software (https://cibersort.stanford.edu/), which has been used previously to accurately predict the frequency of immune cells in various types of tumor tissues [13, 14]. Only those cases with an empirical *P* value < 0.05 using this software (*n* = 170), which indicated a reliable estimation of immune cell infiltration, were used for further survival analysis (details in Materials and Methods). In addition, we analyzed PD- L1 expression in the same tumors. CD8^+^ T cell infiltration or PD-L1 expression alone did not predict differences in survival (Figure 1A, 1B). When CD8^+^ T cell infiltration and PD-L1 expression were analyzed together, patients with tumors having low CD8^+^ T cell infiltration and high PD-L1 expression (CD8^+^ T^lo^PD-L1^hi^) fared significantly worse than patients with tumors demonstrating low CD8^+^ T cell infiltration and low PD-L1 expression (CD8^+^ T^lo^PD-L1^lo^, *P* = 0.039), and approached significantly worse than patients with tumors demonstrating high CD8^+^ T cell infiltration and high PD- L1 expression (CD8^+^ T^hi^PD-L1^hi^, *P* = 0.064), and high CD8^+^ T cell infiltration and low PD-L1 expression (CD8^+^ T^hi^PD-L1^lo^, *P* = 0.066, Figure 1C). Together, this suggests that coupling of PD-L1 expression and the presence of CD8^+^ T cells is required for improved prediction of outcomes.

### Development of established antigenic pancreatic tumors that model the CD8^+^ T^lo^PD-L1^hi^ phenotype

**Figure 2:**
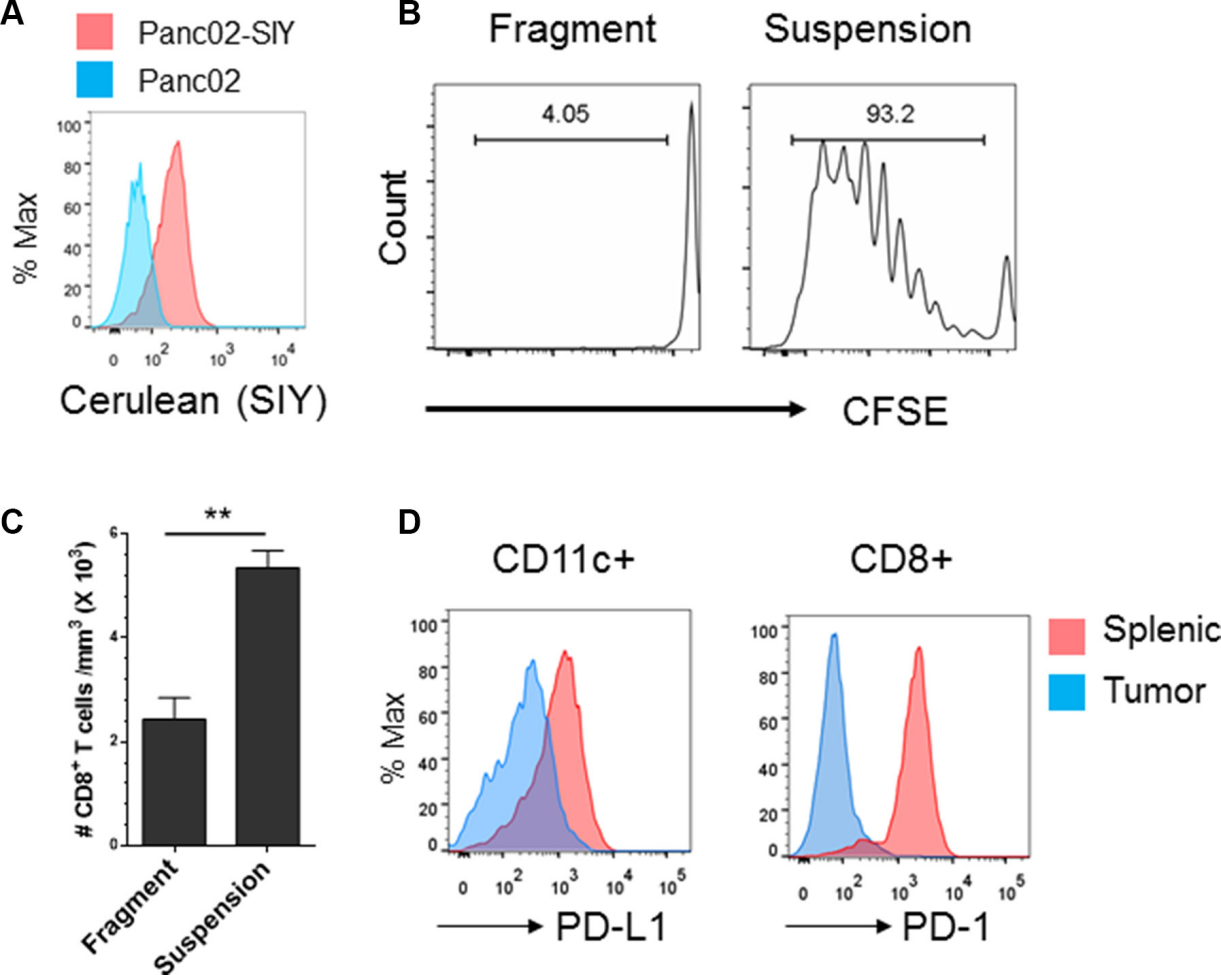
Development of an established syngeneic pancreatic tumor model that mimics the CD8^+^ T^lo^ PD-L1^hi^ phenotype in pancreatic cancer (**A**) Flow cytometric analysis of SIY-cerulean expression of Panc02-Cerulean-SIY^lo^ (Panc02-SIY) cells. Parent Panc02 cell were used as a control. (**B**) Fragment tumors failed to induce T cell priming in draining lymph nodes (DLNs). Fragment tumors were established as described in Materials and Methods. CFSE labeled 2C T cells were injected i.v. into mice bearing fragment tumors or tumors raised from suspension Panc02-SIY cells. Five days later, DLNs were analyzed for CD8^+^ T cell proliferation. (**C**) CD8^+^ T cell density in tumors generated from Panc02-SIY suspension cells and those from fragment tumors. When the size reached 200–250 mm^3^, tumors were processed for flow analysis of CD8^+^ T cells. Cell numbers were determined by flow cytometry analysis. The amount of total CD8^+^ cells were divided by the volume of tumor to determine cell density. Presented data were the summary of one experiment with three mice per group, representing three independent experiments. (**D**) PD-L1 Expression on splenic and tumor infiltrating CD45^+^CD11c^+^ cells (left) and PD-1 expression on splenic and tumor-infiltrating CD45^+^CD8^+^ T cells (right) were measured by flow cytometry respectively. Error bars are mean ± S.E.M. **P < 0.01 (unpaired student's *t*-test).

Since CD8^+^ T^lo^PD-L1^hi^ predicted worse survival in pancreatic cancer, we sought to develop a tumor model that in part mimicked pancreatic cancer with a poorly inflamed phenotype. Since inoculums of cancer cells in suspension induce massive apoptosis and release of antigen that result in artificially primed T cells due to the transplantation process, we generated established tumors arising from inoculums of transplanted tumor fragments that avoided these artifacts of cell injection (Supplementary Figure 1A). To track anti-tumor immune responses, we engineered the C57BL/6 pancreatic cancer cell line Panc02 to express a SIYRYYGL (SIY) antigen fused a to Cerulean fluorescent reporter protein (Figure 2A). The SIY antigen induces strong CD8^+^ T cell responses in C57BL/6 mice [15, 16]. Established tumors arising from inoculums of tumor fragments failed to induce T cell priming in draining lymph nodes (DLNs) as measured by CFSE-labeled 2C T cells that recognize the SIY antigen. By contrast, established tumors arising from suspension cells induced CD8^+^ T cell proliferation in the DLNs (Supplementary Figure 1B and Figure 2B). Established tumors arising from fragments caused significantly less CD8^+^ T cell infiltration compared to tumors derived from suspension cells as measured by flow cytometry (Figure 2C). While Panc02-SIY tumor cells did not have very high PD-L1 expression (Supplementary Figure 2), tumor-infiltrating dendritic cells and CD8^+^ T cells expressed high levels of PD-L1 and PD-l respectively (Figure 2D).

### Vaccination or checkpoint blockade is ineffective against established tumors with a CD8^+^ T^lo^PD-L1^hi^ phenotype

**Figure 3:**
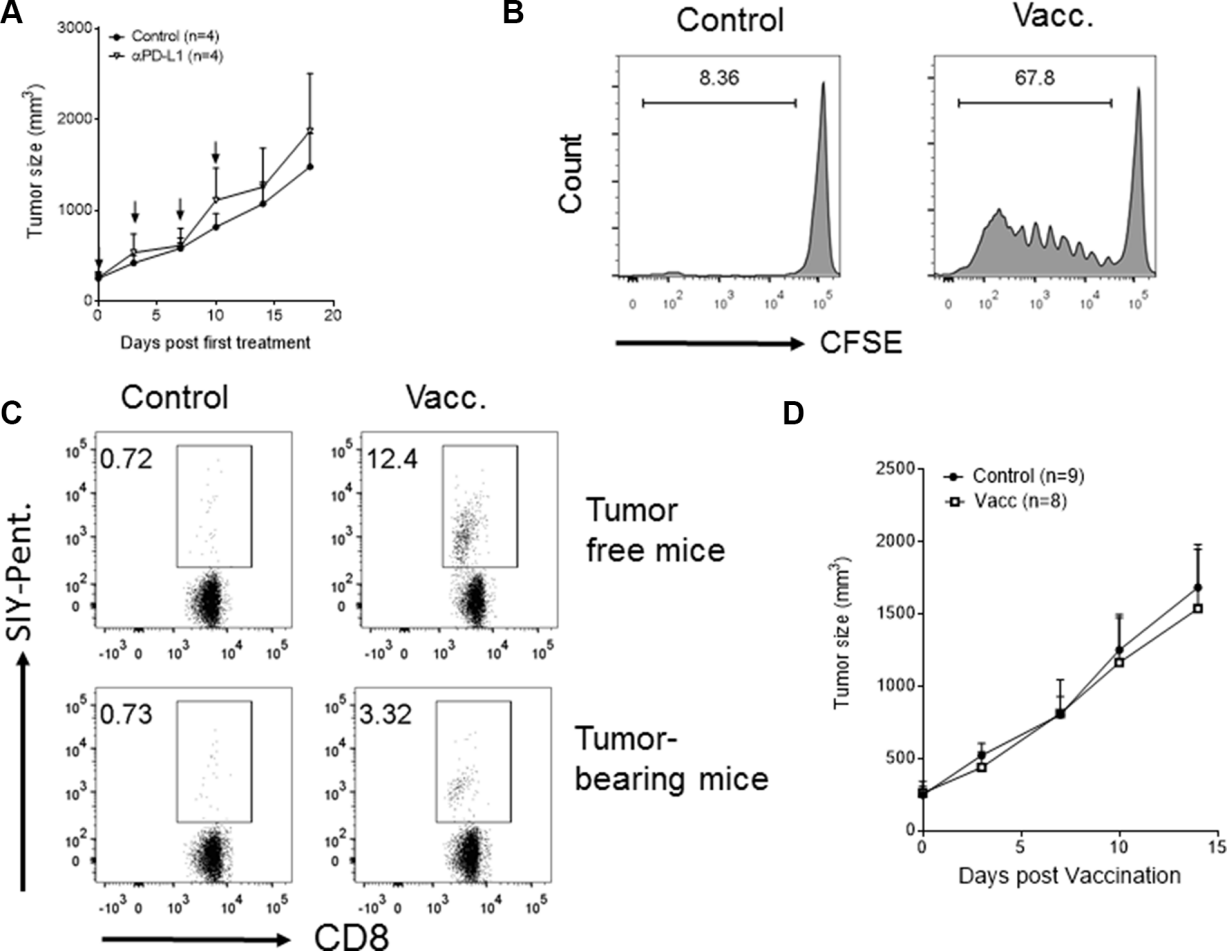
PD-L1 blockade- or vaccination-based immunotherapies fail to cause regression of established tumors with the phenotype of low CD8^+^ T cell infiltrates and high PD-L1 expression (**A**) Tumor growth curves of Panc02-SIY fragment tumor with or without anti-PD-L1 antibody treatment. Anti-PD-L1 antibody was given via intraperitoneal injection with 200 mg/mice at the arrow-indicated time points for a total of 4 doses. (**B–D**) Mice bearing established fragment tumors (Panc02-SIY) were left untreated or vaccinated subcutaneously near the tumor with 2 × 10^6^ MC57-SIY cells (Vacc.). (B) Vaccination induced T cell priming in DLNs was determined by injection of exogenous CFSE-labelled 2C T cells as described in Figure 2B. Events were gated on CD8^+^ T cells. (C) Eight days post vaccination, peripheral blood was stained for flow cytometric analysis of CD8 and the SIY-pentamer. Figures were gated on live CD45^+^CD8^+^ cells. Numbers on the graph indicated percentage of SIY-pentamer-positive cells among all CD8^+^cells. (D) Tumor growth curves of Panc02-SIY fragment tumors with or without MC57-SIY vaccine. Error bars are mean ± S.E.M.

As low CD8^+^ T cell infiltration is associated with poor response to immunotherapy [4, 17–19], we assessed the sensitivity of established Panc02-SIY tumors arising from inoculums of tumor fragments to immunotherapy. PD-L1 blockade failed to inhibit the growth of established Panc02-SIY tumors (Figure 3A), paralleling clinical observations where anti-PD-L1 therapy was ineffective in pancreatic cancer patients [2]. Lack of response to PD-L1 blockade might be attributed to the lack of strong priming. We hypothesized that this lack of strong priming could be due to immunologic ignorance and could be improved with vaccination. Indeed, SIY vaccination induced proliferation of transferred 2C T cells in the DLNs of tumor-bearing mice (Figure 3B) as well as an endogenous anti-SIY immune responses (Figure 3C). However, vaccination against SIY failed to inhibit the growth of Panc02-SIY tumors (Figure 3D). Failure of the tumor to respond significantly to vaccination or checkpoint blockade in this model is consistent with clinical observations of patients with “cold” cancers, which usually fail to respond to vaccine or checkpoint blockade individually.

### Antigen-specific vaccination plus IR converts established tumors from a CD8^+^ T^lo^ to a CD8^+^ T^hi^ phenotype

**Figure 4:**
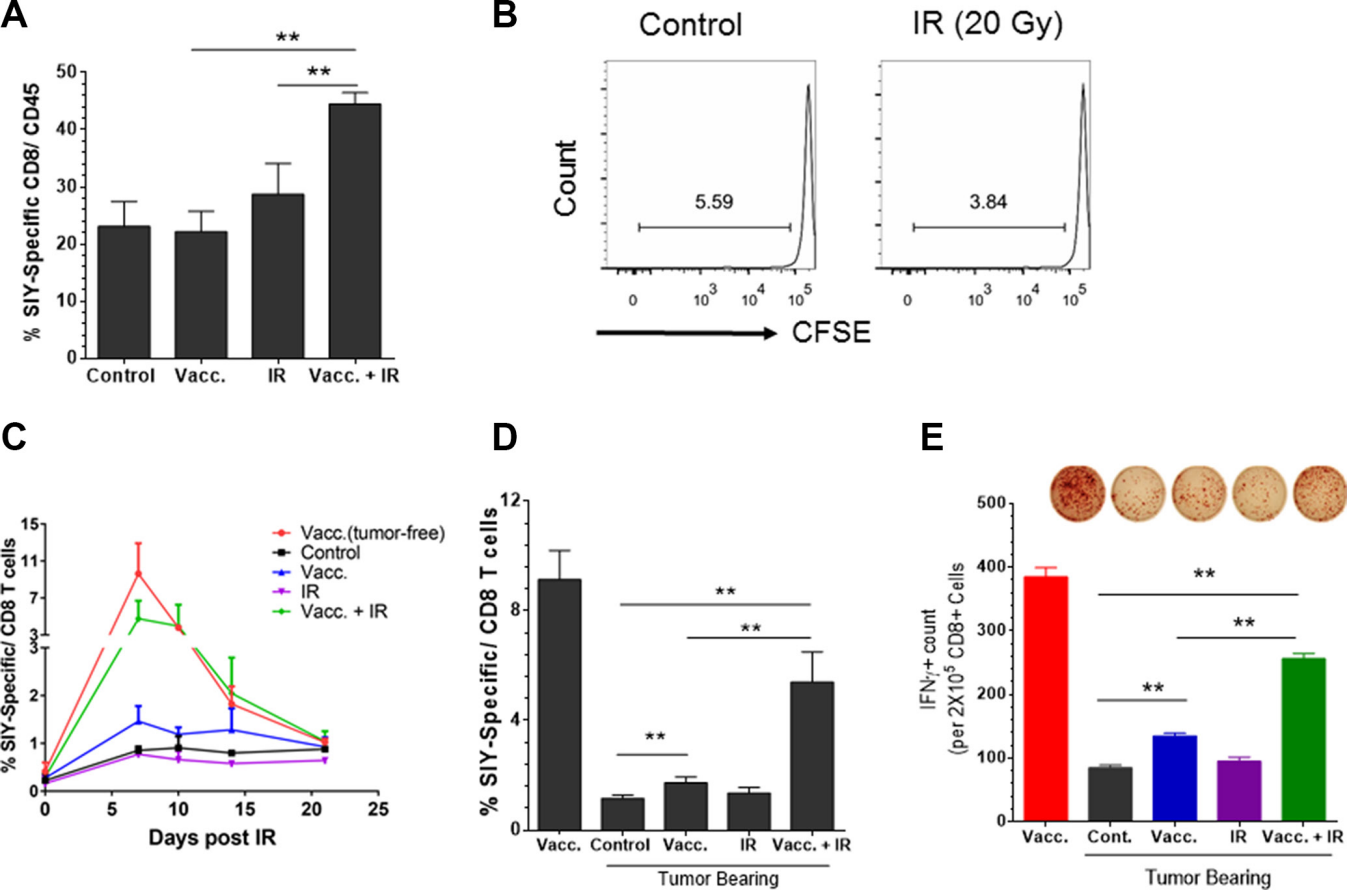
Antigen-specific vaccination plus local radiation convert the established tumor from low level to high level of CD8^+^ T cell infiltration Panc02-SIY fragment tumor-bearing mice were left untreated (control) or received vaccination of 2 × 10^6^ MC57-SIY cells (Vacc.), 20 Gy local ionizing radiation (IR) or vaccination plus IR, respectively. (**A**) Fragment tumors were harvested and processed for flow cytometric analysis of cell surface markers and SIY-Pentamer staining. Quantitative data of the percentage of SIY-specific T cells among all CD8^+^ T cells are presented. (**B**) IR alone fails to prime T cells in DLNs. Panc02-SIY fragment tumor were left untreated or received 20 Gy local IR. CFSE labeled 2C T cells were injected via the tail vein. Five days later, DLNs were analyzed for CD8^+^ T cell proliferation. (**C**) Panc02-SIY fragment tumor-bearing mice were left untreated (control) or received the indicated treatments. Peripheral blood (PB) was processed for flow cytometric analysis for CD8 and the SIY-pentamer at the indicated time points. Eight days post treatment, PB, DLNs were used for following studies: (**D**) PB was evaluated by flow cytometric analysis for CD8 and the SIY-pentamer; (**E**) CD8^+^ T cells were isolated from DLNs and incubated with or without the SIY peptide and irradiated naive splenocytes. The culture systems were applied to IFNγ ELISPOT assay. Lymph node cells from at least three mice in each group were pooled together to obtain one sample. Representative images (upper) and Quantitative data (lower) were presented. Error bar are mean ± S.E.M. *P* values were generated by student's *t*-test, *P < 0.05, **P < 0.01.

We hypothesized that IR could break the tumor barrier, reduce tumor-induced immune suppression, and increase the T cell response against tumor antigens. Since established CD8^+^ T^lo^PD-L1^hi^ tumors demonstrate a poor response to checkpoint blockade, strategies to increase T cell infiltration into tumors may lead to an improved response to PD-L1 blockade. We next evaluated whether adding IR improved T cell infiltration into these tumors, as IR has been shown to alter the immune infiltrate within the tumor microenvironment [9–12]. Established fragment tumors received vaccination, local IR (20 Gy) or combination treatment. We analyzed tumor-infiltrating T cells by flow cytometry and observed that the combination of IR and vaccine promoted infiltration of antigen-specific CD8^+^ T cells into the tumor, as suggested by the increased percentage of SIY-specific CD8^+^ T cells (Supplementary Figure 3 and Figure 4A). The effect of IR on T cell infiltration was not likely due to stimulation of antigen presentation in the DLN, as radiation alone failed to induce 2C T cell proliferation in the DLNs (Figure 4B). Nevertheless, compared to either treatment alone, vaccination and IR increased SIY-specific CD8^+^ T cell response in the peripheral blood (Figure 4C, 4D) and induced a larger amount of IFNγ-producing CD8^+^ T cells in the DLN as measured by ELISPOT (Figure 4E). Taken together, our data suggest that IR might break tumor immune barriers and significantly enhances CD8^+^ T cell infiltration into tumors which was unlikely due to the additive effect of antigen release by irradiated cancer cells.

### IR alters the expression of chemokines and enhances influx of CD8^+^ T cells in the tumor microenvironment

**Figure 5:**
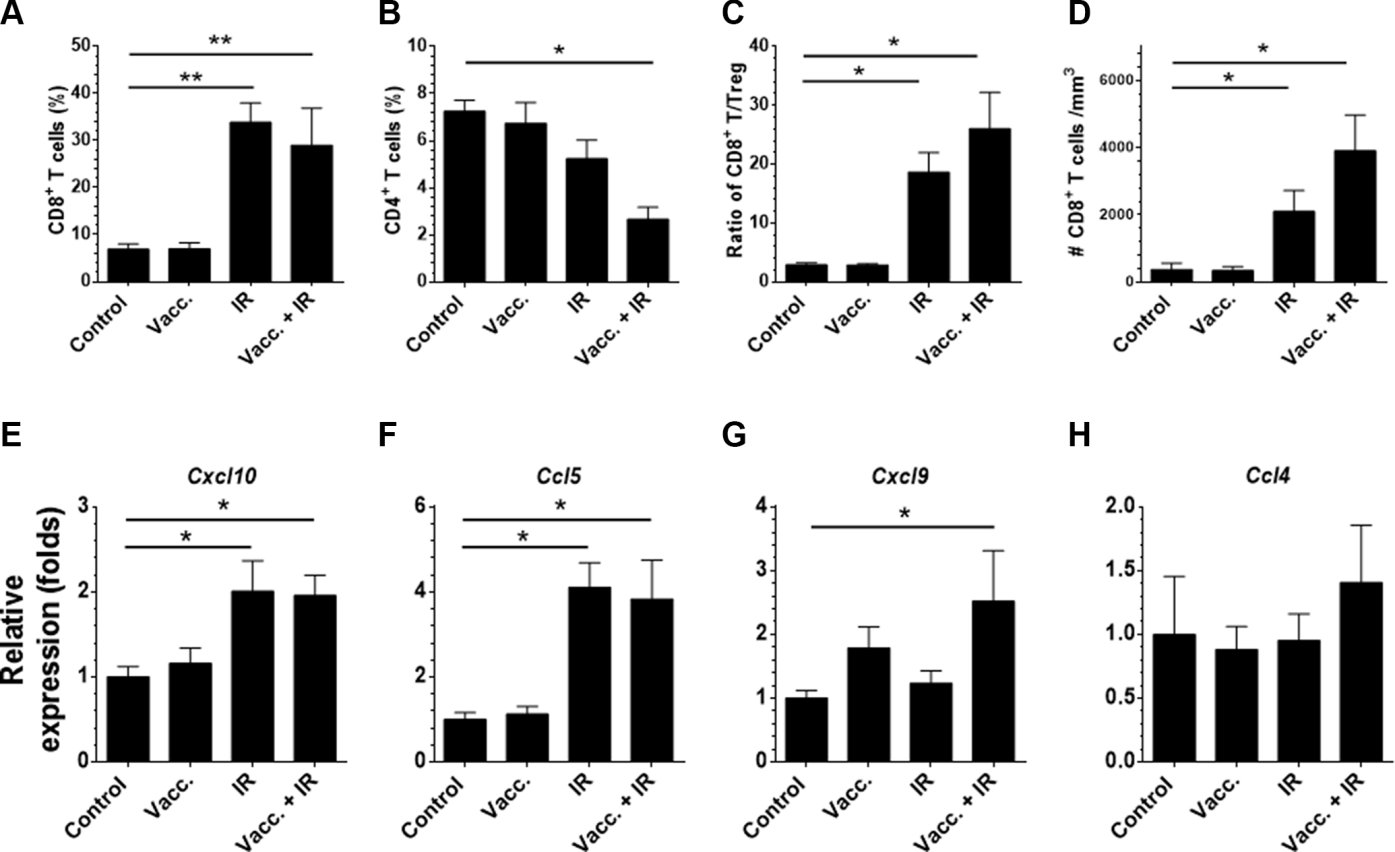
Local radiation alters chemokine expression and preferentially enhances CD8^+^ T cell over Treg infiltration in the fragment tumors Panc02-SIY fragment tumor-bearing mice were left untreated or received one of following treatments: vaccination of 2 × 10^6^ MC57-SIY cells (Vacc.), local IR (20 Gy) or a combination. Eight days post treatment, tumors were removed and processed for flow cytometric analysis. Summary of the percentage of CD8^+^ T cells (**A**), CD4^+^ T cells (**B**) among CD45^+^ cells were presented. The ratio of infiltrating CD8^+^ T over Treg (**C**) and the overall density of CD8^+^ T in the tumor (**D**) were summarized. Eight days post treatment, tumors were removed from the mice and lysed using the Trizol reagent followed by RNA extraction and quantitative PCR analysis of indicated genes (**E–H**). Error bar are mean ± S.E.M. *P* values were generated by student's *t*-test, *P < 0.05, **P < 0.01. (Unpaired student's *t*-test).

To further study whether IR can break the tumor barriers and allow vaccine to increase T cell infiltration into tumors, we assessed if IR enhanced factors that facilitated T cell trafficking in tumors or reduced immune suppression in tumors [10]. First, we analyzed the distribution of immune cells in established tumors after radiotherapy and/or vaccination. As measured by flow cytometry, IR did not affect the percentage of regulatory T cells or myeloid-derived suppressor cells (MDSCs, see Supplementary Figure 4). By contrast, we observed a dramatic increase in CD8^+^ T cells and a decrease in CD4^+^ T cells in the tumors treated with IR (Supplementary Figure 5 and Figure 5A, 5B). There was an increase in the ratio of CD8^+^ T/Treg cells and in the density of CD8^+^ T cells in tumors treated with IR (Figure 5C, 5D). Since IR did not prime SIY-specific responses, increased T cell infiltration was not simply due to increased T cell priming and more likely was due to induction of chemokine expression. We next assessed IR-induced changes in chemokine and cytokine expression that may enhance T cell infiltration [10]. As assessed by qRT-PCR on tumor fragments, IR treatment increased expression of CXCL10 and CCL5, but not CCL4 (Figure 5E–5H). It was reported in another tumor model that a low dose of IR (8 Gy) could upregulate Fas expression on tumor cells [20]. In our system, however, we did not observe a significant change in Fas expression after IR treatment (Supplementary Figure 6). These findings indicate that IR promotes CD8^+^ T cell infiltration by breaking a physical barrier and upregulating chemokines, leading to an increase of the CD8^+^ T/Treg ratio in tumors. In this model, radiation improved infiltration of effector T cells without a concomitant increase in infiltration of immune suppressive cells.

### Administration of an anti-PD-L1 antibody dramatically improves the therapeutic efficacy of IR and vaccination

**Figure 6:**
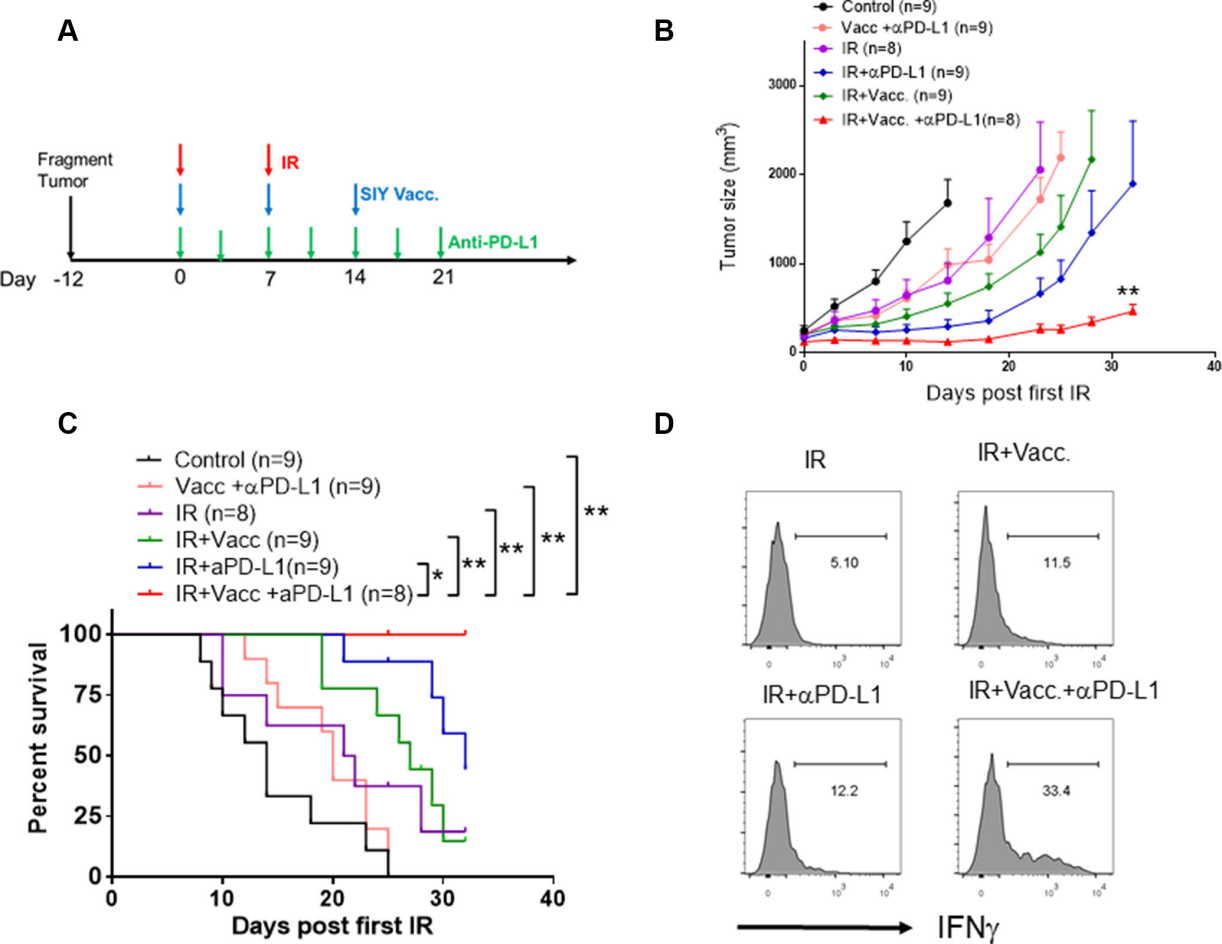
Administration of anti-PD-L1 antibody significantly improves the therapeutic efficacy of vaccination plus IR (**A**) Scheme of the design of treatments strategies. IR: tumor-bearing mice received the first dose of local IR (20 Gy) when the tumor was established (Day 0), and the second dose of IR (15 Gy) on Day 7. Vacc: tumor-bearing mice received vaccination of 2 × 10^6^ MC57-SIY cells on the same day of IR (first dose). Subsequently, mice received 2 subcutaneous doses of boosted vaccine (10 μg SIY peptide plus 20 μg poly I: C for each dose) on Day 7 and Day 21. For PD-L1 blockade, mice received 200 μg/mice anti-PD-L1 antibody i.p. twice a week till Day 21. (**B**) Tumor growth curve of Panc02-SIY fragment tumors which were untreated or received indicated treatments. Error bars are mean ± S.E.M. **P < 0.01, compared with each of other four groups (Two-way ANOVA). (**C**) Overall survival analysis of Panc02-SIY fragment tumor-bearing mice receiving indicated treatments. *P < 0.05, **P < 0.01, Mantel-Cox test. (**D**) Panc02-SIY fragment-bearing mice received indicated treatments. 14 days post first dose of local IR, mice were sacrificed for flow cytometric analysis of IFNγ-producing CD8^+^ cells.

Given that we observed a strong T cell response in the fragment tumor with combination treatment of IR and vaccination, we hypothesized that this combination could restrict tumor growth. However, combination of IR and vaccination demonstrated only a limited T cell-mediated tumor regression (Figure 6A, 6B). Since tumor-infiltrating dendritic cells in Panc02-SIY tumors also had high levels of PD-L1 expression, we hypothesized that checkpoint blockade may also be required even with the increased T cell infiltration observed with tumors treated with IR plus vaccine. Indeed, the combination of anti-PD-L1, IR and vaccination significantly inhibited tumor growth (Figure 6B) and survival of tumor-bearing mice (Figure 6C). This improvement was accompanied by the increase of IFNγ-producing CD8^+^ T cells in the tumors (Figure 6D). Therefore, these results suggest that radiation stimulated the recruitment of vaccine-primed T cells while anti-PD-L1 therapy protected these T cells from local immune suppression. This may be a promising strategy for converting a non-T cell-inflamed tumor to a T cell-inflamed tumor.

## DISCUSSION

Non-T cell-inflamed (“cold”) tumors fail to respond to immunotherapy using checkpoint inhibitors. Specifically, pancreatic cancer has a dismal prognosis and often responds poorly to immunotherapy [1, 2, 21]. We demonstrated that pancreatic cancers with an immune phenotype of low CD8^+^ T cell infiltration and high PD-L1 expression were associated with worse clinical outcomes compared to cancers with high CD8^+^ T cell infiltration and low PD- L1 expression. We established a murine cancer model to study this adverse “cold” phenotype. We then developed an effective approach to control the growth of these aggressive tumors using a combination of radiotherapy, vaccination and checkpoint blockade. Our findings not only provide a model to study “cold” tumors, but also demonstrate a clinically-applicable role for radiotherapy in treating “cold” cancer types by promoting T cell infiltration that improves the efficacy of tumor vaccines and check point inhibitors. This is in contrast to strategies relying upon the direct cytotoxic effects of radiotherapy which have been relatively ineffective in pancreatic cancer, when used alone or with chemotherapy [6–8].

Despite the promising efficacy of immune checkpoint blockade in some types of cancer, monotherapy with PD-1 and CTLA-4 blockade has not proved effective in many cancer types, including pancreatic cancer [1, 2]. In line with this, it has been reported that subsets of melanoma bearing minimal T cell infiltration also respond poorly to checkpoint blockade [3–5]. Notably, the presence and absence of tumor-infiltrating lymphocytes (TILs) and PD-L1 expression were recently used to stratify the tumor microenvironment into four types, each of which may demonstrate a distinct response to checkpoint blockade and require individualized treatment strategies [22, 23]. This newly proposed classification, however, was based on studies in melanoma patients and needs to be validated in other cancer types. It is also notable that the definition of PD-L1 positivity and the presence of TILs was variable and limited by experimental techniques [22, 23]. Interestingly, we demonstrated that neither CD8^+^ T cell infiltration nor PD-L1 expression alone was associated with overall survival of pancreatic cancer patients in TCGA database. However, a combined phenotype of low CD8^+^ T cell infiltration and high expression of PD-L1 (CD8+ T^lo^PD-L1^hi^) predicted a worse clinical outcome. Our experimental fragment murine model, which mimicked this adverse phenotype, received minimal benefit from antigen vaccination or PD-L1 blockade alone. This finding suggests that patients with “cold” cancers with the CD8^+^ T^lo^PD-L1^hi^phenotype may benefit from additional treatments in combination with immunotherapy to achieve sufficient treatment efficacy.

The fragment tumor model we report here is a powerful tool to study immunotherapy in “cold” cancers. When comparing this model to others in the literature [24, 25], we find that this model has several benefits. First, our fragment tumor model accurately mimics the adverse clinical phenotype of CD8^+^ T^lo^PD-L1^hi^. In our model, very few CD8^+^ T cells were present in the tumor, accompanied by the lack of SIY-specific T cells in the blood and lymph nodes of tumor-bearing mice. Additionally, our fragment tumors appeared to be completely resistant to the treatment with anti-PD-L1 antibody alone, which is consistent with the clinical observation that “cold” pancreatic cancer is refractory to checkpoint blockade monotherapy [1, 2]. Second, the incorporation of the immunodominant SIY rejection antigen into cancer cells provided an advantage in the tracking of antigen expression, antigen presentation, and the tumor-specific immune response. Third, by establishing the model in a flank tumor, we can very simply monitor the growth of the tumor, treat with local irradiation without use of complicated radiation equipment or excessive radiation-induced toxicity, and easily extract the tumor and draining lymph node for evaluation. Thus, the model is an excellent means of studying the immune microenvironment of the tumor.

Our study demonstrates that irradiation of “cold” pancreatic cancer increased T cell infiltration and improved the efficacy of immunotherapy for established tumors. Vaccines and other immunotherapies are often ineffective against established tumors as evidenced by previous observations where adoptively-transferred antigen-specific transgenic T cells failed to reject tumors growing for more than 7 days, even after 100-fold increase in T cells transferred [26]. Similarly, our vaccination strategies failed to impact the growth of established pancreatic tumors despite immunization against the immunodominant SIY antigen. Even though established tumors may induce tolerogenic states that blunt immune responses, vaccination against SIY still generated an antigen-specific T cell response in more than 2% of circulating CD8^+^ T cells. By contrast, SIY vaccination failed to stimulate additional infiltration of T cells into tumors suggesting that accessibility of the tumor to CD8^+^ T cells was the limiting step for immune-mediated tumor regression. We demonstrate that IR, a clinically applicable modality for the treatment of pancreatic cancer, also stimulates T cell infiltration into tumors to sensitize pancreatic tumors to immunotherapy. Klug et al. previously noted that low dose irradiation (2 Gy) increased T cell infiltration by reprogramming of tumor-associated macrophages [27]. However, 2 Gy as one or two doses is rarely employed clinically. The 20 Gy single dose irradiation used in our study has clinical relevance, as successful stereotactic body radiotherapy (SBRT) at similar doses has been reported in pancreatic cancer in order to increase local control and achieve cytoreduction [28, 29]. IR has been reported to induce immunological cell death that releases antigen and other signals to stimulate the immune system [30], and may serve as an *in situ* vaccine to elicit T cell immunity [31]. IR may further modulate the immune response by inducing up-regulation of MHC class I molecules, leading to increased tumor recognition by cytotoxic T cells [32]. However, we showed that radiation-induced damage alone is unable to activate endogenous or exogenous T cells in DLNs. We did see a slight increase in the density of SIY-specific T cells in the tumor after IR, suggesting that IR alone may release the antigen to the tumor stroma but not to lymph nodes, a finding consistent with a previous tumor fragment study [33]. Additionally, we found that IR treatment induces an influx of CD8^+^ T cells into the tumor accompanied by elevated expression of CXCL10 and CCL5 in the tumor, both of which are among the signature genes highly expressed in “T cell-inflamed” tumors [34]. This is also consistent with reports that IR promotes immune cell recruitment through increased expression of chemokines [10, 35]. Noteworthy, it was recently reported that epigenetic modulation of CXCL9 and CXCL10 promoted tumor T cell infiltration and enhanced efficacy of PD-L1 blockade [36]. In future studies, it will be interesting to investigate whether IR treatment upregulates these chemokines via altering their epigenetic status. Furthermore, vaccination plus IR generates a much stronger T cell response in the periphery than vaccination alone. A high ratio of CD8^+^ T cells over Treg in the tumor suggests that part of the IR effect may be due to the elimination of some of the immunosuppressive factors. Although radiotherapy is widely used to treat cancers, several trials suggest that radiotherapy does not improve survival in pancreatic cancer [6–8]. Our findings reveal that IR could synergize with immunotherapy to convert cancers with an unfavorable CD8^+^ T^lo^PD-L1^hi^ phenotype to the favorable CD8^+^ T^hi^PD-L1^lo^ phenotype.

We recognize that there are several limitations in our study. First, the streategy depends on a live cell vaccine expressing a model immunodominant antigen. We observed mice carefully after injection of the vaccine; the inoculums completely regressed in all mice observed and no tumors resulted from the vaccine in these immunocompetent mice. It is noteworthy that irradiated tumor vaccines are used in clinical trials in pancreatic cancer [21, 37, 38]. The identification of a sufficiently immunogenic antigen has been a major obstacle for the development of a clinically-effective cancer vaccine [39], though several potential vaccines (such as Muc1 and mesothelin) have reached human trials [21, 37, 38]. It is also noted that viral antigens (such as HPV E6/E7) have been used for vaccine development. In addition, novel neoantigens can be identified by combination of liquid chromatography–mass spectrometry (HPLC-MS) and sequencing. We anticipate that in the future, our model can be applied and validated using these cancer vaccines. Second, it is conceivable that another antigen besides SIY may elicit an immune response against the tumor. However, as we saw SIY-specific T cell activity, this is not likely. The SIY epitope has high MHC binding affinity and causes immunological rejection in several tumor models. The SIY model allowed us to track CD8^+^ T cell responses to cancer expressing this antigen [16]. Additionally, vaccination with MC-57 cells expressing SIY induced SIY-specific CD8^+^ T cell responses but was unlikely to cross-immunize and/or protect against other non-specific antigens potentially shared with Panc02 cells based on previous studies [40].

In conclusion, we report here a new model for the study of cancer biology with an adverse “cold” immunophenotype. Our data demonstrate the potential utility of combining radiotherapy, vaccination, and checkpoint blockade in pancreatic cancer and other “cold” cancers. We propose a novel therapeutic strategy using vaccination and local radiation to induce antigen-specific T cell infiltration in the tumor, and ultimately blockade of PD-L1-mediated immunosuppression in the tumor microenvironment to lead to immunologic control of tumor growth. We believe that further validation of our model with regard to tumor T cell infiltration and expression of PD-L1 will yield a patient selection strategy which will allow the application of IR and anti-PD-L1 therapy for patients with “cold” cancers, and will lead to improved outcomes.

## MATERIALS AND METHODS

### Mice

Six- to eight-week-old C57BL/6 mice were purchased from Harlan and used for the indicated experiments. 2C T cell-receptor (TCR) transgenic mice and OT-1 TCR transgenic mice were purchased from The Jackson Laboratory. All mice were maintained under specific pathogen-free conditions and used in accordance with the animal experimentation guidelines set by the Institute of Animal Care and Use Committee of the University of Chicago.

### Tumor growth and treatment

Panc02 and MC57-SIY cells were kindly provided by Dr. Hans Schreiber (University of Chicago). Panc02 cells were infected by retrovirus with pMFG (SIY)_3_-Cerulean as described [41, 42]. After infection, Panc02-SIY-Cerulean cells were FACS-sorted for low expression of SIY-Cerulean to generate the Panc02-Cerluean-SIY^lo^ (Panc02-SIY) cell line. The cell lines were authenticated by a short tandem repeat profile (IDEXX Bioresearch) within the last 6 months. Cells were cultivated and used within 20 passages.

For tumors from suspension cells, Panc02 or Panc02-SIY cells were trypsinized, washed with media, and were injected subcutaneously into C57BL/6 mice (1 × 10^6^ cells/100 μl/mouse). For the fragment tumor model, harvested Panc02-SIY cells were injected subcutaneously into OT-1 T cell receptor transgenic mice (1 × 10^6^ cells/100 μl/mouse). 4–6 weeks post inoculation, established Panc02- SIY tumors were excised, divided into 1–2mm fragments, and implanted subcutaneously into naive C57BL/6 mice. Tumor volumes were measured along three orthogonal axes (*a, b*, and *c*) and calculated as tumor volume = *abc*/2. Mice with tumors greater than 1500 mm^3^ in volume were euthanized in accordance with the animal protocol. These mice were counted as dead mice in survival analyses.

Fragment tumor-bearing mice were allowed 12 days before any treatment in order to reestablish viable tumors. For local IR treatment, mice were irradiated using an x-ray generator (PCM 1000; Pantak) at the doses indicated by each experiment. Each mouse was protected with a lead cover with only tumor exposed, allowing local irradiation. In some experiments, mice received a 2^nd^ dose of local IR on day 7 after the initial IR dose. For vaccination treatments, on the same day of IR (first dose), 2 × 10^6^ live MC57-SIY cells were injected subcutaneously onto the back of mice, close to the fragment tumor. In some experiments, mice were injected with 2 subcutaneous doses of boosted vaccine (10 mg SIY peptide plus 20 μg poly I:C for each dose) on Day 7 and Day 21 post IR. For PD-L1 blockade, mice received 200 μg/mice anti-PD-L1 antibody (10F.9G2, BioXcell) intraperitoneally at indicated time points.

### Flow cytometric analysis

To obtain single-cell suspensions, tumor tissues were digested with 1mg/ml Collagenase IV (Sigma) and 0.2 mg/ml DNase I (Sigma) for 45 min at 37°C. Single-cell suspensions were incubated with 2.4G2 to block antibody binding to the Fc receptors and then subsequently stained with conjugated antibodies: anti-CD45.2 (clone 104), anti-CD90.2 (clone 30-H12), anti-CD8a (clone 53–6.7), anti-CD11c (clone HL3), anti-CD11b (clone M1/70), anti-Ly6C (clone HK1.4), anti-Ly6G (clone 1A8), anti-CD4 (clone GK1.5), anti-PD-1 (clone 29F.1A12), anti-PD-L1 (clone MIH5) and SIY-Pentamer (Proimmune). In the indicated experiments for further identification of regulatory T cells, after surface marker staining, cells were fixed and used for intracellular staining of anti-Foxp3 (clone FJK-16s) antibody according the manufacturer's instruction (eBioscience). For intracellular staining of IFNγ, after surface marker staining, cells were fixed and stained with anti-IFNγ antibody (clone XMG1.2). For CFSE dilution analysis, at the indicated time points, DLN cells were harvested and labeled with conjugated anti-CD45.2 and anti-CD8a antibodies before analysis by FACS. All other purified and fluorescently-labeled monoclonal antibodies were purchased from BioLegend. Samples were analyzed on an LSRFortessa Flow Cytometer (BD) and data were analyzed with FlowJo Software (TreeStar).

### ELISPOT

Eight days after treatment, tumor DLNs were removed and CD8^+^ T cells were purified with EasySep Mouse CD8α Positive Selection Kit (StemCell). 2 × 10^5^ CD8^+^ T cells were incubated with 1 × 10^5^ irradiated (12 Gy) splenocytes from naive mice in the presence or absence of 1 μg/ml SIY peptide for 48 hours. ELISPOT assays were performed to detect the cytokine spots of IFN-γ according to product protocol (Millipore).

### Quantitative RT-PCR

Tumors were harvested eight days after the indicated treatments. Quantitative Real-time PCR was conducted on cDNA prepared from DNase I-treated RNA extracted from whole tumor fragments. Gene specific primers from the genes were synthesized and the sequences were provided as followed. The expression was normalized to the housekeeping gene *B2m* (beta-2 macroglobulin). *B2m* FW 5′- TTC TGG TGC TTG TCT CAC TGA-3′, *B2m* RV 5′-CAG TAT GTT CGG CTT CCC ATT C-3′; *Cxcl9* FW, 5′- TAG GCA GGT TTG ATC TCC GT -3′, *Cxcl9* RV, 5′- CGA TCC ACT ACA AAT CCC TCA -3′; *Cxcl10* FW, 5′- CCT ATG GCC CTC ATT CTC AC -3′, *Cxcl10* RV, 5′- CTC ATC CTG CTG GGT CTG AG-3′; *Ccl4* FW, 5′-GAA ACA GCA GGA AGT GGG AG -3′, *Ccl4* RV, 5′- CAT GAA GCT CTG CGT GTC TG -3′; *Ccl5* FW, 5′- CCA CTT CTT CTC TGG GTT GG -3′, *Ccl5* RV, 5′- GTG CCC ACG TCA AGG AGT AT -3′. Reactions were run on the ABI/Prism 7300 (Applied Biosystems), in a final volume of 25 μl with 2.5 μM of the forward and reverse primers using 2x SYBR green Master Mix (Applied Biosystems) containing polymerase. Cycling conditions were a single denaturing step at 95°C for 15 min followed by 40 cycles of 94°C for 15 s and 60°C for 1 min.

### CIBERSORT and database analysis

Gene expression and clinical data of pancreatic cancer patients were collected from TCGA database (*n* = 183). The online analytic tool, CIBERSORT (Cell type Identification By Estimating Relative Subsets Of known RNA Transcripts), was used following the manual provided by the developers (https://cibersort.stanford.edu/). CIBERSORT is a recently reported computational approach that used gene expression profiles to estimate relative fractions of diverse cell subsets complex tissues, including tumor [13, 14]. LM22, a validated a leukocyte gene signature matrix [13], was used here as a gene signature matrix. LM22 contains 547 genes that distinguish 22 human hematopoietic cell phenotypes, including CD8+ T cells, Th1, Th2, naïve and memory B cells, plasma cells, natural killer cells and myeloid subsets. The gene expression data from the pancreatic cancer patients in TCGA database was input as a Mixture file. CIBERSORT also used Monte Carlo sampling to generate an empirical *P* value for the deconvolution [13]. Only those cases with an empirical *P* value < 0.05 using this software, which indicated a reliable estimation of immune cell infiltration, were used for further survival analysis.

### Statistical analysis

Tumor growth curves were assessed by repeated-measure ANOVA. Survival curves were compared by log rank (Mantel-COX) test. Differences between two groups were analyzed by a two-tailed unpaired student *t* test. All statistics analyses were performed using GraphPad Prism 6.0 or SAS9.4 (SAS Institute Inc.). *P <* 0.05 denotes differences that are statistically significant.

## SUPPLEMENTARY MATERIALS



## References

[1] Royal RE, Levy C, Turner K, Mathur A, Hughes M, Kammula US, Sherry RM, Topalian SL, Yang JC, Lowy I, Rosenberg SA (2010). Phase 2 trial of single agent Ipilimumab (anti-CTLA-4) for locally advanced or metastatic pancreatic adenocarcinoma. J Immunother.

[2] Brahmer JR, Tykodi SS, Chow LQ, Hwu WJ, Topalian SL, Hwu P, Drake CG, Camacho LH, Kauh J, Odunsi K, Pitot HC, Hamid O, Bhatia S (2012). Safety and activity of anti-PD-L1 antibody in patients with advanced cancer. N Engl J Med.

[3] Gajewski TF (2015). The Next Hurdle in Cancer Immunotherapy: Overcoming the Non-T-Cell-Inflamed Tumor Microenvironment. Semin Oncol.

[4] Gajewski TF, Schreiber H, Fu YX (2013). Innate and adaptive immune cells in the tumor microenvironment. Nat Immunol.

[5] Harlin H, Meng Y, Peterson AC, Zha Y, Tretiakova M, Slingluff C, McKee M, Gajewski TF (2009). Chemokine expression in melanoma metastases associated with CD8+ T-cell recruitment. Cancer Res.

[6] Gillen S, Schuster T, Meyer Zum Buschenfelde C, Friess H, Kleeff J (2010). Preoperative/neoadjuvant therapy in pancreatic cancer: a systematic review and meta-analysis of response and resection percentages. PLoS Med.

[7] Kim EJ, Ben-Josef E, Herman JM, Bekaii-Saab T, Dawson LA, Griffith KA, Francis IR, Greenson JK, Simeone DM, Lawrence TS, Laheru D, Wolfgang CL, Williams T (2013). A multi-institutional phase 2 study of neoadjuvant gemcitabine and oxaliplatin with radiation therapy in patients with pancreatic cancer. Cancer.

[8] Kharofa J, Tsai S, Kelly T, Wood C, George B, Ritch P, Wiebe L, Christians K, Evans DB, Erickson B (2014). Neoadjuvant chemoradiation with IMRT in resectable and borderline resectable pancreatic cancer. Radiother Oncol.

[9] Barker HE, Paget JT, Khan AA, Harrington KJ (2015). The tumour microenvironment after radiotherapy: mechanisms of resistance and recurrence. Nat Rev Cancer.

[10] Burnette B, Weichselbaum RR (2013). Radiation as an immune modulator. Semin Radiat Oncol.

[11] Formenti SC, Demaria S (2013). Combining radiotherapy and cancer immunotherapy: a paradigm shift. J Natl Cancer Inst.

[12] Lee Y, Auh SL, Wang Y, Burnette B, Wang Y, Meng Y, Beckett M, Sharma R, Chin R, Tu T, Weichselbaum RR, Fu YX (2009). Therapeutic effects of ablative radiation on local tumor require CD8+ T cells: changing strategies for cancer treatment. Blood.

[13] Newman AM, Liu CL, Green MR, Gentles AJ, Feng W, Xu Y, Hoang CD, Diehn M, Alizadeh AA (2015). Robust enumeration of cell subsets from tissue expression profiles. Nat Methods.

[14] Gentles AJ, Newman AM, Liu CL, Bratman SV, Feng W, Kim D, Nair VS, Xu Y, Khuong A, Hoang CD, Diehn M, West RB, Plevritis SK (2015). The prognostic landscape of genes and infiltrating immune cells across human cancers. Nat Med.

[15] Engels B, Chervin AS, Sant AJ, Kranz DM, Schreiber H (2012). Long-term persistence of CD4(+) but rapid disappearance of CD8(+) T cells expressing an MHC class I-restricted TCR of nanomolar affinity. Mol Ther.

[16] Spiotto MT, Yu P, Rowley DA, Nishimura MI, Meredith SC, Gajewski TF, Fu YX, Schreiber H (2002). Increasing tumor antigen expression overcomes “ignorance” to solid tumors via crosspresentation by bone marrow-derived stromal cells. Immunity.

[17] Fridman WH, Pages F, Sautes-Fridman C, Galon J (2012). The immune contexture in human tumours: impact on clinical outcome. Nat Rev Cancer.

[18] Leen AM, Rooney CM, Foster AE (2007). Improving T cell therapy for cancer. Annu Rev Immunol.

[19] Pardoll DM (2012). The blockade of immune checkpoints in cancer immunotherapy. Nat Rev Cancer.

[20] Chakraborty M, Abrams SI, Coleman CN, Camphausen K, Schlom J, Hodge JW (2004). External beam radiation of tumors alters phenotype of tumor cells to render them susceptible to vaccine-mediated T-cell killing. Cancer Res.

[21] Le DT, Wang-Gillam A, Picozzi V, Greten TF, Crocenzi T, Springett G, Morse M, Zeh H, Cohen D, Fine RL, Onners B, Uram JN, Laheru DA (2015). Safety and survival with GVAX pancreas prime and Listeria Monocytogenes-expressing mesothelin (CRS-207) boost vaccines for metastatic pancreatic cancer. J Clin Oncol.

[22] Smyth MJ, Ngiow SF, Ribas A, Teng MW (2015). Combination cancer immunotherapies tailored to the tumour microenvironment. Nat Rev Clin Oncol.

[23] Teng MW, Ngiow SF, Ribas A, Smyth MJ (2015). Classifying Cancers Based on T-cell Infiltration and PD-L1. Cancer Res.

[24] Logsdon CD, Arumugam T, Ramachandran V (2015). Animal Models of Gastrointestinal and Liver Diseases. The difficulty of animal modeling of pancreatic cancer for preclinical evaluation of therapeutics. Am J Physiol Gastrointest Liver Physiol.

[25] Herreros-Villanueva M, Hijona E, Cosme A, Bujanda L (2012). Mouse models of pancreatic cancer. World J Gastroenterol.

[26] Hanson HL, Donermeyer DL, Ikeda H, White JM, Shankaran V, Old LJ, Shiku H, Schreiber RD, Allen PM (2000). Eradication of established tumors by CD8+ T cell adoptive immunotherapy. Immunity.

[27] Klug F, Prakash H, Huber PE, Seibel T, Bender N, Halama N, Pfirschke C, Voss RH, Timke C, Umansky L, Klapproth K, Schakel K, Garbi N (2013). Low-dose irradiation programs macrophage differentiation to an iNOS(+)/M1 phenotype that orchestrates effective T cell immunotherapy. Cancer cell.

[28] Schellenberg D, Goodman KA, Lee F, Chang S, Kuo T, Ford JM, Fisher GA, Quon A, Desser TS, Norton J, Greco R, Yang GP, Koong AC (2008). Gemcitabine chemotherapy and single-fraction stereotactic body radiotherapy for locally advanced pancreatic cancer. Int J Radiat Oncol Biol Phys.

[29] Chang DT, Schellenberg D, Shen J, Kim J, Goodman KA, Fisher GA, Ford JM, Desser T, Quon A, Koong AC (2009). Stereotactic radiotherapy for unresectable adenocarcinoma of the pancreas. Cancer.

[30] Pilones KA, Vanpouille-Box C, Demaria S (2015). Combination of radiotherapy and immune checkpoint inhibitors. Semin Radiat Oncol.

[31] Lugade AA, Moran JP, Gerber SA, Rose RC, Frelinger JG, Lord EM (2005). Local radiation therapy of B16 melanoma tumors increases the generation of tumor antigen-specific effector cells that traffic to the tumor. J Immunol.

[32] Reits EA, Hodge JW, Herberts CA, Groothuis TA, Chakraborty M, Wansley EK, Camphausen K, Luiten RM, de Ru AH, Neijssen J, Griekspoor A, Mesman E, Verreck FA (2006). Radiation modulates the peptide repertoire, enhances MHC class I expression, and induces successful antitumor immunotherapy. J Exp Med.

[33] Zhang B, Bowerman NA, Salama JK, Schmidt H, Spiotto MT, Schietinger A, Yu P, Fu YX, Weichselbaum RR, Rowley DA, Kranz DM, Schreiber H (2007). Induced sensitization of tumor stroma leads to eradication of established cancer by T cells. J Exp Med.

[34] Messina JL, Fenstermacher DA, Eschrich S, Qu X, Berglund AE, Lloyd MC, Schell MJ, Sondak VK, Weber JS, Mule JJ (2012). 12-Chemokine gene signature identifies lymph node-like structures in melanoma: potential for patient selection for immunotherapy?. Sci Rep.

[35] Matsumura S, Wang B, Kawashima N, Braunstein S, Badura M, Cameron TO, Babb JS, Schneider RJ, Formenti SC, Dustin ML, Demaria S (2008). Radiation-induced CXCL16 release by breast cancer cells attracts effector T cells. J Immunol.

[36] Peng D, Kryczek I, Nagarsheth N, Zhao L, Wei S, Wang W, Sun Y, Zhao E, Vatan L, Szeliga W, Kotarski J, Tarkowski R, Dou Y (2015). Epigenetic silencing of TH1-type chemokines shapes tumour immunity and immunotherapy. Nature.

[37] Ramanathan RK, Lee KM, McKolanis J, Hitbold E, Schraut W, Moser AJ, Warnick E, Whiteside T, Osborne J, Kim H, Day R, Troetschel M, Finn OJ (2005). Phase I study of a MUC1 vaccine composed of different doses of MUC1 peptide with SB-AS2 adjuvant in resected and locally advanced pancreatic cancer. Cancer Immunol Immunother.

[38] Le DT, Brockstedt DG, Nir-Paz R, Hampl J, Mathur S, Nemunaitis J, Sterman DH, Hassan R, Lutz E, Moyer B, Giedlin M, Louis JL, Sugar EA (2012). A live-attenuated Listeria vaccine (ANZ-100) and a live-attenuated Listeria vaccine expressing mesothelin (CRS-207) for advanced cancers: phase I studies of safety and immune induction. Clin Cancer Res.

[39] Gaudernack G (2006). Prospects for vaccine therapy for pancreatic cancer. Best Pract Res Clin Gastroenterol.

[40] Prehn RT, Main JM (1957). Immunity to methylcholanthrene-induced sarcomas. J Natl Cancer Inst.

[41] Schietinger A, Arina A, Liu RB, Wells S, Huang J, Engels B, Bindokas V, Bartkowiak T, Lee D, Herrmann A, Piston DW, Pittet MJ, Lin PC (2013). Longitudinal confocal microscopy imaging of solid tumor destruction following adoptive T cell transfer. Oncoimmunology.

[42] Engels B, Engelhard VH, Sidney J, Sette A, Binder DC, Liu RB, Kranz DM, Meredith SC, Rowley DA, Schreiber H (2013). Relapse or eradication of cancer is predicted by peptide-major histocompatibility complex affinity. Cancer cell.

