# Supplementary material for: Combination of radiotherapy and vaccination overcomes checkpoint blockade resistance
Source: Oncotarget. 2016 Jun 7;7(28):43039–51. doi: 10.18632/oncotarget.9915 (PMC5190006; doi:10.18632/oncotarget.9915)
Supplement: Supplementary file 1 [file oncotarget-07-43039-s001.pdf]

Combination of radiotherapy and vaccination overcomes checkpoint blockade resistance

Supplementary Materials

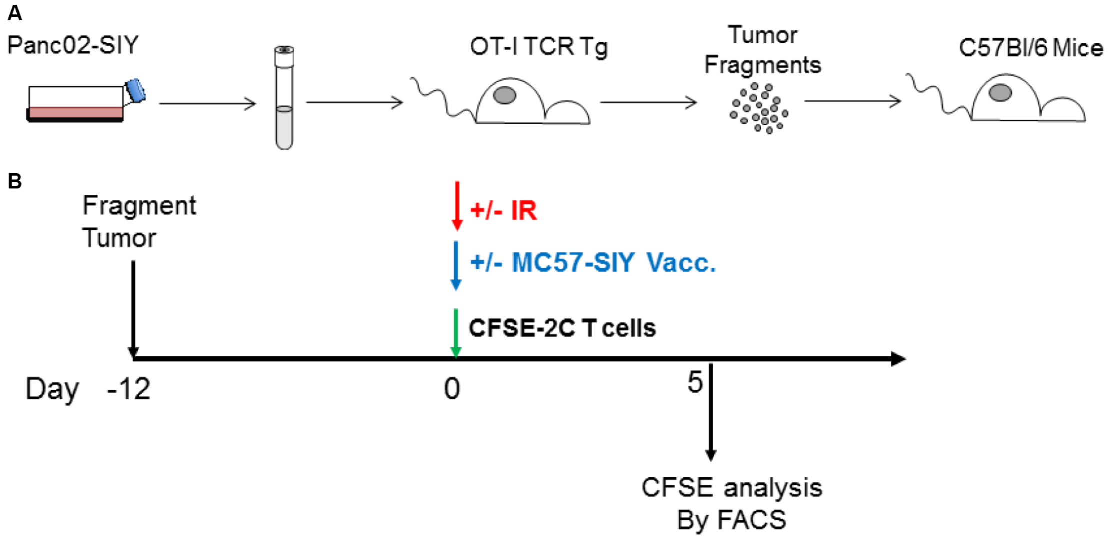

Supplementary Figure S1: (**A**) Scheme of the approach for establishing the fragment tumor model. Panc02- SIY cells were grown *in vitro* prior to subcutaneous injection of the cells in suspension to OT-1 TCR transgenic mice. 4–6 weeks post inoculation, established Panc02-SIY tumors were excised, divided into 1–2 mm fragments, and implanted subcutaneously into naive C57Bl/6 mice. Tumor growth was observed for 10–14 days prior to treatments. (**B**) Scheme of the strategy to detect T cell priming in DLNs by the administration of exogenous CFSE-labeled 2C T cells. Fragment tumor were established as described in (A) and received indicated treatment and/or 5×10^6 CFSE-labelled naive 2C T cells on Day 0 (12 days post fragment transplantation). On day 5, DLNs were removed for flow cytometric analysis of CFSE intensity.

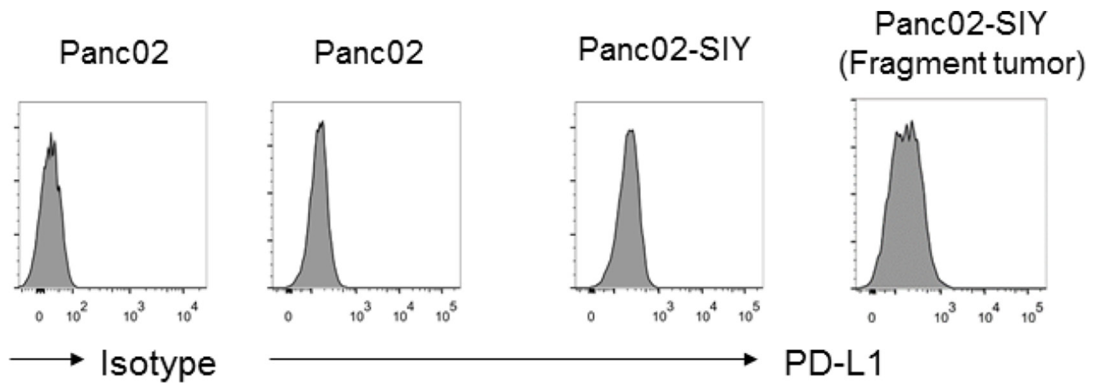

Supplementary Figure S2: Panc02-SIY tumor cells demonstrate low PD-L1 expression. Panc02-SIY and the parental cell line Panc02 were used for flow cytometric analysis of PD-L1 expression. Panc02-SIY fragment-tumor-bearing mice were left untreated. On day 21 post transplantation, tumors were removed from the mice and cut into small pieces. Resulting portions were culture in DMEM medium with 10% FBS for 3–4 days followed by flow cytometric analysis of PD-L1 expression on $CD45^-$ cells.

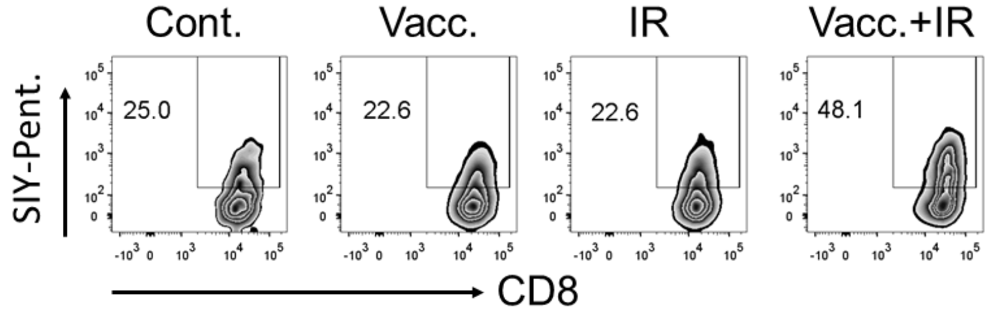

Supplementary Figure S3: Antigen-specific vaccination plus local radiation increased percentage of antigen-specific CD8+ T cells. Panc02-SIY fragment tumor-bearing mice were left untreated (control) or received vaccination of 2 × 10^6 MC57-SIY cells (Vacc.), 20 Gy local ionizing radiation (IR) or vaccination plus IR, respectively. Fragment tumors were harvested and processed for flow cytometric analysis of cell surface markers and SIY-Pentamer staining. Representative dot plots of SIY-specific T cells among all $CD8^+$ T cells are presented.

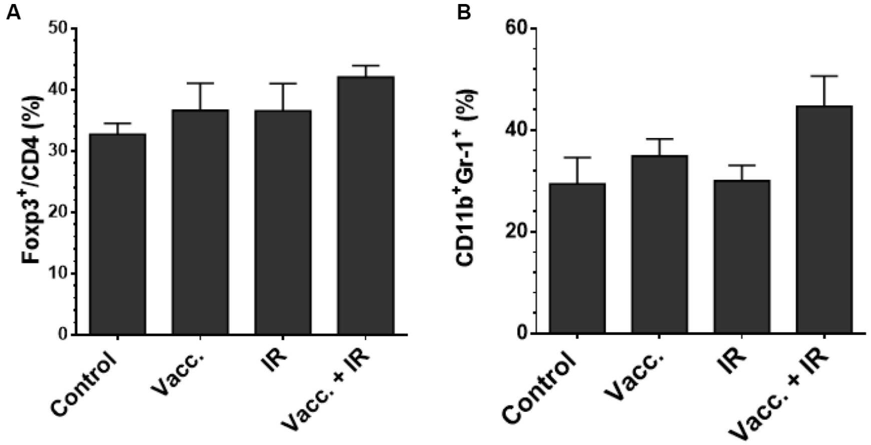

Supplementary Figure S4: Percentage of Tregs and MDSCs in the tumors post treatments. Panc02-SIY fragment-tumor-bearing mice were left untreated or received one of following treatments: vaccination with 2 × 10^6 MC57-SIY cells, local IR (20 Gy) or a combination of both. Eight days post treatment, tumors were removed and processed for flow cytometric analysis of immunosuppressive cells, including (**A**) $Foxp3^+$ cells among total $CD4^+$ cells (**B**) $CD11b^+GR\text{-}1^+$ MDSCs. Error bars are mean ± S.E.M. Presented data are the summary of an experiment with at least three samples in each group. The experiments were repeated at least twice.

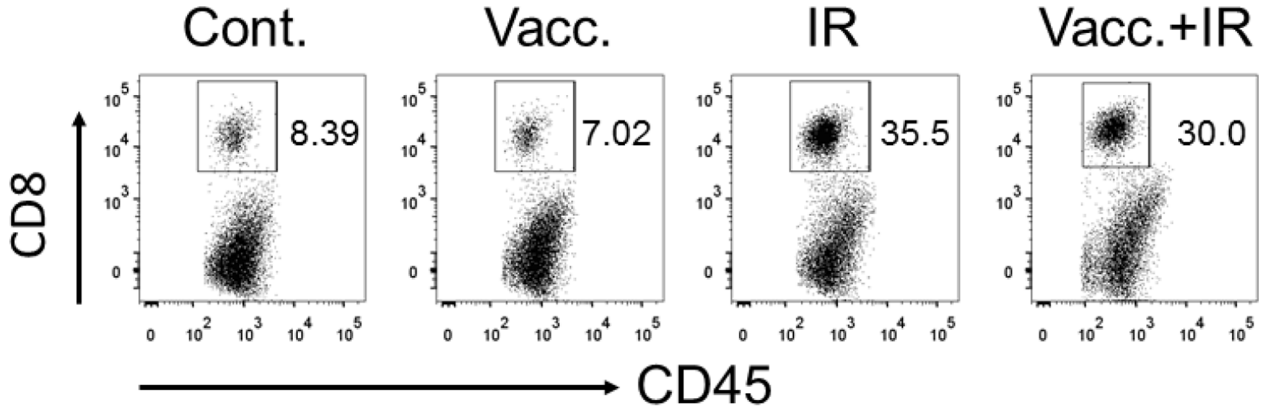

Supplementary Figure S5: Local radiation enhances CD8⁺ T cell infiltration in the fragment tumors. Panc02-SIY fragment tumor-bearing mice were left untreated or received one of following treatments: vaccination of 2×10^6 MC57-SIY cells (Vacc.), local IR (20 Gy) or a combination. Eight days post treatment, tumors were removed and processed for flow cytometric analysis. Representative dot plots of $CD8^+$ T cells among all $CD45^+$ cells are presented.

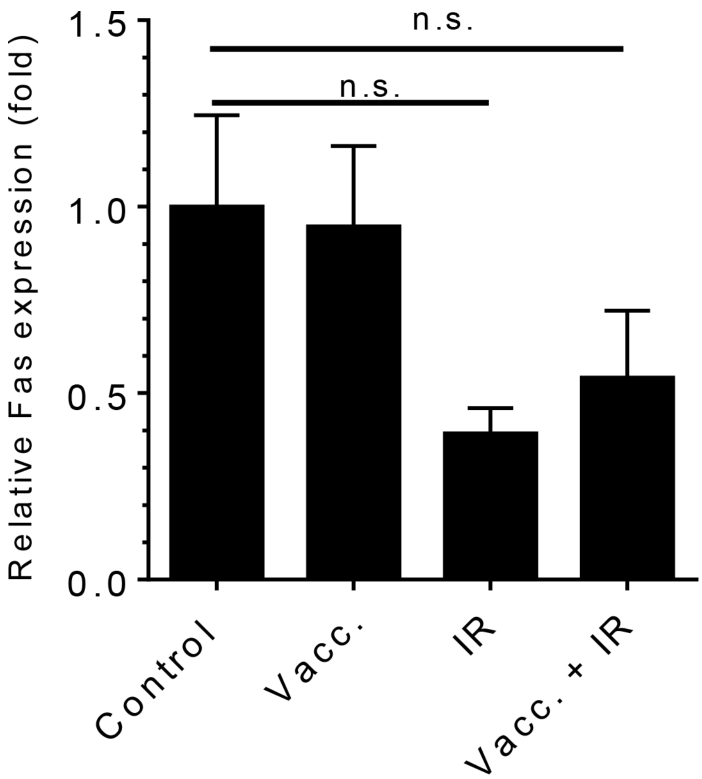

Supplementary Figure S6: Fas expression in Panc02-SIY fragment tumor. Panc02-SIY fragment tumor-bearing mice were left untreated or received one of following treatments: vaccination of 2×10^6 MC57-SIY cells (Vacc.), local IR (20 Gy) or a combination. Eight days post treatment, tumors were removed from the mice and lysed using the Trizol reagent followed by RNA extraction and quantitative PCR analysis of Fas expression. n.s.: Not statistically significant. (Unpaired student's *t*-test).
